# Comprehensive management of synchronous colorectal liver metastases at a high-volume center: a propensity score-matched analysis

**DOI:** 10.1007/s13304-025-02348-1

**Published:** 2025-08-21

**Authors:** Agostino M. De Rose, Elena Panettieri, Andrea Campisi, Viviana Esposito, Francesco Belia, Maria Vellone, Francesco Ardito, Felice Giuliante

**Affiliations:** Hepatobiliary Surgery, Fondazione Policlinico Universitario A. Gemelli IRCCS, Università Cattolica del Sacro Cuore, L.Go A. Gemelli 8, 00168 Rome, Italy

**Keywords:** Synchronous colorectal liver metastases, Hepatectomy, High-volume referral center, Surgical approach, Multidisciplinary management

## Abstract

The optimal management of synchronous colorectal liver metastases (CLM) remains debated, particularly regarding the role of centralized care. This study aimed to assess the impact of comprehensive management at a high-volume center on perioperative and long-term outcomes in patients undergoing curative-intent hepatectomy for synchronous CLM. We retrospectively analyzed 613 patients treated from 2010 to 2021 at a tertiary referral center. Patients were categorized as exclusively internally managed (*n* = 273) or partially externally managed (*n* = 340). Propensity score matching (PSM) was performed to minimize bias. Exclusively internally managed patients were characterized by a greater rate of rectal cancer and a higher liver tumor burden. Overall, major morbidity was 11.0% and 90-day mortality was 0.6%, with no significant difference between groups. Median overall survival (OS) was 68 months. Independent predictors of worse OS included rectal cancer, advanced T-stage, nodal positivity, > 6 chemotherapy cycles, major hepatectomy, and R1 margins. After PSM (*n* = 428), exclusively internally managed patients showed improved 5-year OS (54.5% vs. 44.6%, *p* = 0.054). Comprehensive multidisciplinary management at a high-volume center may improve long-term outcomes in patients with synchronous CLM, particularly those with more advanced disease. Timely referral and consistent multidisciplinary tumor board evaluation are essential to optimize outcomes and guide personalized treatment strategies.

## Introduction

Colorectal cancer (CRC) is the third most common cancer worldwide in terms of incidence, and the second leading cause of cancer-related mortality [1]. Along with the decreased incidence of metachronous colorectal liver metastases (CLM), the occurrence of synchronous CLM has been increasing over time, probably due to improved imaging techniques leading to earlier diagnosis [2]. Synchronous CLM are associated with worse survival compared to metachronous metastases, reflecting their inherent poor biology. [3, 4]. Current guidelines on the surgical management of patients with synchronous CLM provide only broad recommendations, and clinical practice is primarily guided by national and international consensus statements [5–7].

Given the complexity of care required, the treatment of synchronous CLM should be centralized in specialized high-volume centers to guarantee a comprehensive multidisciplinary approach and select the most appropriate sequence of treatment, integrating complex multi-organ surgery with systemic chemotherapy [8]. While ideal, this approach is not always adopted as many patients receive care, at least partially, at non-specialized institutions. The treatment strategies for patients with synchronous CLM differ markedly between institutions. High-volume centers tend to implement more aggressive and coordinated multidisciplinary approaches, which have been associated with better clinical outcomes compared to those reported by lower-volume facilities [9].

The aim of the study was to assess clinical management of patients with synchronous CLM, analyzing the effect of treatment at a high-volume cancer center since diagnosis. Additionally, perioperative and long-term outcomes were evaluated.

## Methods

### Patient selection

Data from all consecutive patients undergoing first curative-intent liver resection for CLM from January 2010 through December 2021 at Fondazione Policlinico Universitario Agostino Gemelli IRCCS, Università Cattolica del Sacro Cuore (Rome, Italy) were retrospectively analyzed. Patients were identified from a prospectively maintained database established at our Hepatobiliary Surgery Unit, that systematically records all hepatic resections performed.

Patients at least 18 years old who underwent first curative-intent hepatectomy were identified. Patients who underwent resection with macroscopic residual tumor were excluded. Patients who underwent two-stage hepatectomy and repeat hepatectomy were included once in the dataset.

### Ethics statement

The study was approved by the institutional review board of Fondazione Policlinico Universitario Agostino Gemelli IRCCS, Università Cattolica del Sacro Cuore.

Data collection and analysis were performed according to institutional guidelines and conformed to the ethical standards of the World Medical Association (Declaration of Helsinki) [10].

### Definitions

Exclusively internally managed patients were those treated at Fondazione Policlinico Universitario Agostino Gemelli IRCCS, Università Cattolica del Sacro Cuore since diagnosis. Partially externally managed patients were those who received part of their care at outside institutions but underwent hepatectomy at our center.

Synchronous CLM were detected within 12 months from diagnosis or surgery of the primary tumor [11]. The Tumor Burden Score (TBS) was calculated on the basis of tumor size and total number of tumors as follows: TBS2 = (maximum diameter)^2^ + (number of tumors)^2^. [12] TBS values were categorized as low (< 3), medium (≥ 3 to < 9), and high (≥ 9) as originally proposed [12]. Colorectal cancer staging was based on the eighth edition of the AJCC Cancer Staging Manual [13].

Type of liver resection (major vs. minor) was based on the Brisbane classification and resection of three or more adjacent segments was classified as major hepatectomy [14]. Parenchyma-sparing resection was preferred whenever feasible, from an oncological and technical standpoint, as it allows for reduced sacrifice of functioning parenchyma while maintaining adequate oncologic radicality [15]. Parenchymal-sparing liver surgery refers to multiple resections of  ≥ 3 CLM optimizing the remnant liver volume. Sequencing of primary tumor resection and CLM resection was classified as simultaneous, primary tumor resection first (classic approach), or CLM resection first (liver-first or reverse approach).

Complications were described according to the Clavien–Dindo classification [16]. Major morbidity refers to Clavien–Dindo grade 3 or greater complications.

Overall survival (OS) was defined as months between surgical resection of CLM and death.

For patients with synchronous extrahepatic disease requiring surgical, interventional, or adjuvant systemic treatment, management was performed entirely at our institution, regardless of initial referral status, as part of a coordinated multidisciplinary approach.

### Preoperative assessment

Preoperative radiologic investigations included abdominal ultrasonography, abdominal computed tomography (CT) scan or magnetic resonance imaging (MRI), and CT scan of the chest. All patients were evaluated by a multidisciplinary liver tumor board (MDLTB) including hepatobiliary and colorectal surgeons, clinical and radiation oncologists, radiologists, and interventional radiologists. Based on our policy, there were no predefined criteria of unresectability with regard to number, size, and CLM laterality. Patients were defined resectable when all disease could be removed leaving an adequate future liver remnant volume. Unresectability was defined by inadequate liver remnant or by the impossibility to remove all CLM either by one- or two-stage procedure. An anticipated risk of R1 (parenchymal and/or vascular) resection was not a contraindication to liver resection, although our strategy was to obtain a tumor-free margin, whenever possible.

### Preoperative chemotherapy

Indications to preoperative chemotherapy were: initially unresectable CLM or marginally resectable CLM (high risk of R1 resection due to number, size, or challenging location of CLM; multiple, synchronous CLM).

Response to chemotherapy was assessed every two months (four cycles of chemotherapy) by the MDLTB up until the achievement of surgical resectability. Response to chemotherapy was determined using the Response Evaluation Criteria in Solid Tumors (RECIST) [17]. In case of initially unresectable CLM, liver resection was performed as soon as resectability was technically possible, without waiting for further radiologic response. In case of borderline resectable CLM, hepatectomy was usually performed after 2 months of chemotherapy.

### Statistical analysis

Quantitative variables were reported as median and interquartile range (IQR). Qualitative variables were reported as absolute and relative frequency (percentage).

Quantitative variables were compared using the Mann–Whitney *U* test, while categorical variables were compared using the Chi-square test.

Survival curves were generated using the Kaplan–Meier method, and difference between curves was assessed with the log-rank test. Univariable and multivariable analyses to identify factors associated with OS were performed using the Cox proportional hazards regression model. A subgroup analysis comparing patients that were exclusively internally managed and patients that were partially externally managed was performed. A 1:1 propensity score matching (PSM) was preformed to balance prognostic factors between the two groups and reduce the impact of confounding factors. The matched variables included: rectal cancer,  ≥ 3, positive lymph nodes, high TBS, and synchronous extrahepatic disease.

All statistical tests were two-sided, and statistical significance was defined as *p* less than 0.05. Analyses were performed using SPSS version 25 (IBM Corporation, Armonk, NY, USA).

## Results

### Study population

Of 613 patients included, 273 (44.5%) were exclusively internally managed, and 340 (55.5%) were partially externally managed. For the entire population, the mean number of CLM was 3 (IQR 1–5) with a rate of bilateral disease of 53.5% and a median larger tumor size of 2.6 cm (IQR 1.6–4). The median TBS was 4.6 and 16.6% of patients had a high TBS. Synchronous extrahepatic disease was detected in 12.6% of patients. Regarding CRC, rectal cancer was diagnosed in 35.8% of cases, while 21.2% of patients had right colon cancer.

After comparing the two patient subgroups, there was no substantial difference in terms of median number of CLM, maximum CLM diameter, *RAS* mutation rate, rate of bilateral CLM, and synchronous extrahepatic disease (Table 1).

**Table 1:** Patient and tumor characteristics of patients who underwent resection of synchronous colorectal liver metastases after exclusively internal management or partially external management

	Entire population (*n* = 613)	Exclusively internal management (*n* = 273)	Partially external management (*n* = 340)	*p* value
**Demographic factors**				
Age, median (IQR), yr	64 (56–70)	64 (56–70)	63 (56–70)	0.334
Men	366 (59.7)	159 (58.2)	207 (60.9)	0.508
CLM factors				
Bilateral	328 (53.5)	150 (54.9)	178 (52.3)	0.510
Number of CLM, median (IQR)^a^	3 (1–5)	3 (1–5)	2 (1–5)	0.154
CLM maximum size, median (IQR), cm^b^	2.6 (1.6–4)	2.7 (1.5–4.1)	2.6 (1.7–4)	0.843
TBS, median (IQR)^c^	4.6 (3–7.2)	4.7 (3.1–7.8)	4.6 (2.9–6.7)	0.162
High TBS, ^c^	99 (16.6)	54 (20.5)	45 (13.5)	*0.022*
Synchronous extrahepatic disease	77 (12.6)	32 (11.7)	45 (13.2)	0.574
**Primary tumor factors**				
Rectal cancer^d^	219 (35.8)	119 (43.6)	100 (29.6)	< *0.001*
Right colon cancer^d^	130 (21.2)	57 (20.9)	73 (21.6)	0.829
T ≥ 3^e^	513 (90.5)	218 (88.3)	295 (92.2)	0.114
Positive lymph nodes^f^	393 (69.7)	168 (68.3)	225 (70.7)	0.528
**Chemotherapy factors**				
Preoperative chemotherapy	545 (88.9)	243 (89.0)	302 (88.8)	0.941
Number of cycles, median (IQR)^g^	8 (5–12)	7.5 (5–12)	8 (5–12)	0.104
Number of cycles > 6 g	323 (61.2)	140 (59.8)	183 (62.2)	0.572
Targeted therapies^h^	395 (74.1)	172 (72.0)	223 (75.8)	0.309
*RAS* mutation^i^	219 (44.4)	96 (44.9)	123 (44.1)	0.864

Italics indicate statistical significance

*IQR* Interquartile range; *BMI* Body mass index; *ASA* American Society of Anesthesiologists; *CLM* Colorectal liver metastases; *TBS* Tumor burden score

Values are number (percentage) unless indicated otherwise

^a^Data missing on number of CLM for 2 patients

^b^Data missing on CLM maximum size for 9 patients

^c^Data missing on TBS for 37 patients

^d^Data missing on primary tumor location for 2 patients

^e^Data missing on T for 46 patients

^f^Data missing on lymph-node status for 49 patients

^g^Data missing on number of chemotherapy cycles for 17 patients

^h^Data missing on targeted therapy for 12 patients

^i^Data missing on *RAS* mutational status for 120 patients

Interestingly, compared with partially externally managed patients, those who were exclusively internally treated more frequently had rectal cancer (43.6% vs. 29.6%; *p* < 0.001), and more frequently presented with a high TBS (20.5% vs. 13.5%; *p* = 0.049).

Preoperative chemotherapy was administered in 88.9% of cases. In particular, 61.2% of patients received > 6 cycles of treatment, with a median 8 cycles (IQR 5–12) being delivered in the overall population and a rate of associated targeted therapies of 74.1%. No significant differences in terms of preoperative chemotherapy administration were noted among the two subpopulations (Table 1).

### Perioperative outcomes

Major hepatectomy was performed in 28.7% of patients, with a rate of two-stage hepatectomy of 7.5%. Parenchymal-sparing liver resection was indicated in 39.0% of patients and a minimally invasive approach was preferred in 14.7% of cases. No significant differences in terms of hepatectomy approaches were noticed between subgroups.

A liver-first approach and a simultaneous resection approach were only adopted in patients exclusively internally managed, respectively, in 16.8% and 30.8% of cases. Among patients that were selected for a liver-first approach, 69.6% had rectal cancer and 50% underwent major hepatectomy. Among patients who underwent simultaneous CLM and CRC resection, 41.7% had rectal cancer and 81.0% required minor liver resection.

Overall, an R1 resection was achieved in 28.2% of hepatectomies. Regarding post-operative outcomes, 90-day mortality was 0.6% and 90-day major complications occurred in 11.0% of patients, with no significant difference between subgroups (Table 2).

**Table 2:** Intraoperative and short-term post-operative outcomes of patients who underwent resection of synchronous colorectal liver metastases after exclusively internal management or partially external management

	Entire population (*n* = 613)	Exclusively internal management (*n* = 273)	Partially external management (*n* = 340)	*p* value
**Intraoperative factors**				
Minimally invasive approach	90 (14.7)	45 (16.5)	45 (13.2)	0.259
Major hepatectomy	176 (28.7)	79 (28.9)	97 (28.5)	0.912
Two-stage hepatectomy	46 (7.5)	25 (9.2)	21 (6.2)	0.164
Parenchymal-sparing hepatectomy	239 (39.0)	108 (39.6)	131 (38.5)	0.795
Liver-first approach	46 (7.5)	46 (16.8)	0 (0)	< *0.001*
Simultaneous colorectal resection	84 (13.7)	84 (30.8)	0 (0)	< *0.001*
Intraoperative transfusion	50 (8.2)	24 (8.8)	26 (7.6)	0.607
R1 resection^a^	178 (28.2)	82 (28.7)	96 (27.8)	0.442
Short-term outcomes				
90-day mortality	4 (0.6)	3 (1.1)	1 (0.3)	0.219
90-day major complications*	67 (11.0)	37 (13.7)	30 (8.8)	0.057

Italics indicate statistical significance

*IQR* Interquartile range

Values are number (percentage) unless indicated otherwise

^a^Data missing on margin status for 7 patients

^*^Excluding post-operative deaths

### Long-term outcomes

**Fig. 1:**
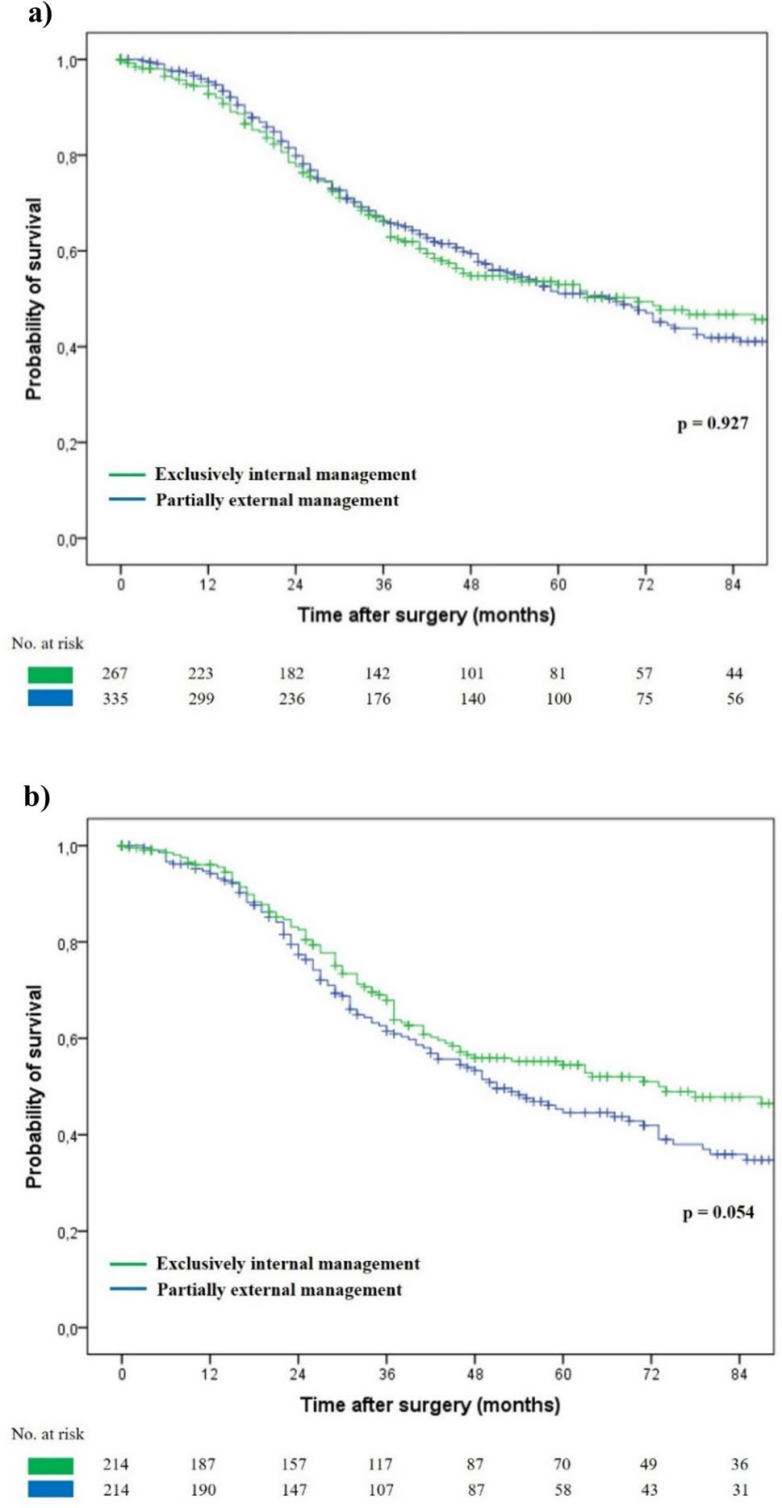
Overall survival of patients who underwent liver resection for synchronous colorectal liver metastases according to type of management: **a** before and **b** after propensity score matching

For the entire population, 1-, 3-, and 5-year OS rates were 94.2%, 66.2%, and 51.9%, respectively. Median OS was 68 months. After subgroup analysis, 1-, 3-, and 5-year OS rates were 92.8%, 66.1%, and 53%, respectively, for exclusively internally managed patients, and 95.3%, 66.2%, and 51.1%, respectively, for partially externally managed patients. Median overall survival was 71 months for exclusively internally managed patients and 67 months for partially externally managed patients (*p* = 0.927) (Fig. 1a). After univariable and multivariable analysis, the following factors were independently associated with OS in the overall population: rectal cancer, T ≥ 3, lymph-node metastases, preoperative chemotherapy > 6 cycles, major hepatectomy, and positive surgical margins (Table 3).

**Table 3:** Multivariable Cox proportional hazards model analysis for overall survival for patients who underwent resection of synchronous colorectal liver metastases

Overall survival						
Factor	Univariable analysis			Multivariable analysis		
	HR	95% CI	*p* value	HR	95% CI	*p* value
CRC factors						
Rectal cancer	1.29	1.01–1.64	*0.040*	1.34	1.01–1.77	*0.039*
T ≥ 3	2.21	1.29–3.80	*0.004*	2.27	1.19–4.33	*0.012*
Positive lymph nodes	1.50	1.13–1.99	*0.005*	1.39	1.02–1.89	*0.040*
Preoperative factors						
High TBS	2.04	1.52–2.75	< *0.001*	1.62	1.16–2.28	*0.005*
Exclusively internal management	0.98	0.77–1.24	0.874	–	–	–
Chemotherapy > 6 cycles	1.64	1.25–2.15	< *0.001*	1.42	1.07–1.89	*0.015*
Operative factors						
Major hepatectomy	1.77	1.38–2.27	< *0.001*	1.42	1.07–1.89	*0.016*
R1 resection	1.71	1.32–2.20	< *0.001*	1.45	1.09–1.92	*0.010*
*RAS* mutation	1.15	0.89–1.49	0.289	–	–	–

Italics indicate statistical significance

*CRC* Colorectal cancer; *HR* Hazard ratio; *CI* Confidence interval

### Patient subgroup analysis after propensity score matching

To minimize bias and balance prognostic differences among the two groups, a 1:1 PSM was performed including biological variables significantly associated with OS (rectal cancer, T ≥ 3, positive lymph nodes, and high TBS), as well as the presence of synchronous extrahepatic disease (Table 4). A total of 428 patients were matched, with 214 patients in each group. Right-sided colon cancer was significantly more frequent in the exclusively internally managed group (23.8% vs. 15.9%, *p* = 0.039), while the distribution of *RAS* mutation, chemotherapy-related variables, and surgical factors, including R1 resection, were comparable between the two matched cohorts. The only significant differences concerning the surgical strategy were between liver-first and simultaneous CLM and CRC resection approaches, that were both solely performed in the exclusively internally managed group. For the entire matched population, 1-, 3-, and 5-year OS rates were 95.1%, 64.7%, and 49.5%, respectively. Median OS was 60 months. After subgroup analysis, 1-, 3-, and 5-year overall survival rates were 96%, 67.9%, and 54.5%, respectively, for exclusively internally managed patients, and 94.2%, 61.5%, and 44.6%, respectively, for partially externally managed patients. Median OS was 73 months for exclusively internally managed patients and 51 months for partially externally managed patients (*p* = 0.054) (Fig. 1b).

**Table 4:** Patient and tumor characteristics of patients who underwent resection of synchronous colorectal liver metastases after exclusively internal management or partially external management after propensity scored matching

	After PSM		
	Exclusively internal management (*n* = 214)	Partially external management (*n* = 214)	*p* value
Matched confounding prognostic factors			0.689
Rectal cancer	78 (36.4)	82 (38.3)	
T ≥ 3	195 (91.1)	197 (92.1)	0.728
Positive lymph nodes	148 (69.2)	154 (72.0)	0.525
High TBS	32 (14.9)	30 (14.0)	0.784
Synchronous extrahepatic disease	30 (14.0)	33 (15.4)	0.682
Other clinically/oncological factors			
Right colon cancer	51 (23.8)	34 (15.9)	*0.039*
* RAS* mutation	76 (35.5)	78 (36.4)	0.756
Chemotherapy factors			
Preoperative chemotherapy	193 (90.2)	191 (89.3)	0.750
Number of cycles > 6	27 (12.6)	28 (13.1)	0.653
Intraoperative factors			
Minimally invasive approach	39 (18.2)	33 (15.4)	0.438
Major hepatectomy	56 (26.2)	63 (29.4)	0.450
Two-stage hepatectomy	19 (8.9)	14 (6.5)	0.365
Parenchymal-sparing hepatectomy	86 (40.2)	76 (35.5)	0.319
Liver-first approach	30 (14)	0	< *0.001*
Simultaneous colorectal resection	66 (30.8)	0	< *0.001*
R1 resection	54 (25.2)	54 (25.2)	1.000

Italics indicate statistical significance

*PSM* propensity score matching; *TBS* tumor burden score

Values are number (percentage) unless indicated otherwise

## Discussion

This study compared outcomes of patients undergoing hepatectomy for synchronous CLM following exclusively internal vs. partially external management at a high-volume hepatobiliary center. Patients who were exclusively internally treated presented with more advanced disease, with a higher frequency of rectal cancer and a higher metastatic liver burden. Regarding the surgical strategy, simultaneous resection of CLM and CRC and a liver-first approach were exclusively adopted among patients treated entirely within our institution. No differences in perioperative morbidity and OS were observed between the two patient groups, despite a higher disease burden and more unfavorable prognostic factors in the exclusively internally treated cohort. However, after applying propensity score matching to balance baseline prognostic characteristics between the groups, exclusively internally treated patients demonstrated a survival advantage that approached statistical significance.

Notably, almost 90% of patients received preoperative chemotherapy, with no significant differences between the two groups. This result is in line with what reported by the Expert Group on OncoSurgery Management of Liver Metastasis (EGOSLIM) in 2015 [11], and in the more recent Multisocietal European Consensus [7], that states that most patients with resectable synchronous CLM should undergo upfront systemic chemotherapy prior to surgical treatment. A recent meta-analysis further supported this sequencing strategy, showing a 12% lower risk of recurrence in favor of preoperative chemotherapy in patients with high-burden disease, despite a higher rate of R1 resection, suggesting that neoadjuvant chemotherapy may mitigate the negative prognostic impact of R1 surgery [18]. Sarkar et al., through a recent analysis of 43,039 patients with liver-only CLM from the NCDB colorectal cancer dataset, demonstrated a correlation between perioperative chemotherapy and superior OS [19].

In terms of surgical strategy, the choice among the three available options, liver-first, colon-first, and simultaneous approach, should be based on liver and colorectal tumor burden and on the evaluation of the surgical risk associated with the planned procedure. To date, the treatment strategy for synchronous CLM relies on a case-by-case evaluation by multidisciplinary expert teams rather than on robust evidence-based data.

Data from the LiverMetSurvey registry on 7360 patients with synchronous CLM showed that 60% of them underwent colorectal resection first, 7.5% liver surgery first, and 32.5% simultaneous resection, a proportion that remained relatively constant over time at around 30% [8]. These results seem aligned with the evidence presented in our paper.

The most important implication of the simultaneous approach is to reduce post-operative morbidity and mortality. A recent review and meta-analysis comparing 2762 patients undergoing simultaneous resection versus 3655 patients undergoing staged resection showed that the simultaneous resection group had a shorter hospital stay and similar OS, but an increased post-operative mortality [20]. The authors concluded that the simultaneous approach should be avoided in patients at high surgical risk due to increased perioperative mortality [20]. The presence of multiple comorbidities was the factor that was more strongly associated with a high risk of post-operative complications, often requiring longer hospital stay and readmission, especially after rectal resection and simultaneous hepatectomy [21]. Similar results were reported by Endo et al., who found a higher complication rate after simultaneous resection in patients with high liver tumor burden, even after propensity score matching [22].

A completely different scenario characterizes the liver-first approach. This “reverse” approach appears particularly appropriate in patients with advanced CLM and rectal cancer with an indication for chemoradiotherapy, that could delay CLM resection and increase the risk of progression to unresectability [23, 24]. In our series, 16.8% of patients underwent a liver-first approach, predominantly among those with rectal cancer (69.6%) and extensive liver involvement, with 50% undergoing major hepatectomy. The proportion of patients with synchronous CLM managed with a liver-first approach in tertiary referral centers ranges from 7 to 40% in the literature, with most of these patients having rectal cancer (approximately 50–60%) or a high hepatic tumor burden [8, 25–28]. It is also important to highlight that rectal cancer is biologically associated with a poorer prognosis, often presenting with a higher hepatic tumor burden, and that patients with rectal cancer are more frequently directed to tertiary referral centers, as confirmed by the characteristics of our study population. Such complexity—simultaneous surgery, rectal cancer, and high hepatic tumor burden—is reflected in an increased, although not statistically significant, morbidity rate in the group of patients treated exclusively internally.

Simultaneous and staged strategies (including primary-first and liver-first approaches) for treating CRC with synchronous CLM are comparable in terms of long-term survival [29, 30]. Our results are consistent with the existing literature, showing a 5-year OS of 52%, with no significant differences between the exclusively internally managed and partially externally managed groups, despite the greater complexity and higher disease burden among the former.

Survival of resected synchronous CLM was strongly influenced by primary tumor-related factors (CRC location, T ≥ 3, lymph-node metastases), CLM-related factors (high TBS), and surgical factors (major hepatectomy, R1 resection). Notably, receiving more than 6 cycles of preoperative chemotherapy was also identified as an independent risk factor for OS, underscoring the need for surgical referral as early as possible to ensure timely intervention. Of note, recent data by Rhaiem et al. emphasized the complex interaction between *RAS* mutational status and resection margin in CLM, suggesting that R0 resection may offer a survival benefit predominantly in *RAS* wild-type tumors [31]. In our matched cohort, the distribution of *RAS* mutation and R1 resection was balanced, further supporting that the improved survival observed in the exclusively internally managed group is not merely attributable to biological differences.

It is pivotal to note that all patients treated at our institution were discussed within a MDLTB. The role of the MDLTB is crucial to tailor the most appropriate treatment strategy for each patient with synchronous CLM [11]. The MDLTB can positively influence clinical decision-making and patient care. Specifically, Oxenberg et al. reported that although 84% of clinicians were initially confident in their treatment plan, a change was recommended in 36% of cases after multidisciplinary discussion, with 72% of these changes being major [32]. In particular, a dedicated MDLTB focused on metastatic CRC has proven to be more effective than a generic tumor board. According to Vallance et al., patients diagnosed and treated in centers with specialized hepatobiliary surgical teams are more likely to undergo timely liver resection and show improved long-term outcomes, with a median survival of 30 months vs. 25 months in centers without hepatobiliary expertise [33]. Viganò et al. also emphasized the advantages of managing CLM in a single specialized center, highlighting that care in hepatobiliary referral units is associated with shorter durations of preoperative chemotherapy, better disease control, fewer surgical procedures due to the feasibility of simultaneous resections, and improved survival, especially when patients are referred early [34].

The main limitation of our study relates to the definition of synchronous CLM. Currently, neither the National Comprehensive Cancer Network (NCCN) [35] nor the European Society for Medical Oncology (ESMO) [36] CRC guidelines provide a standardized definition of synchronous CLM, which can create challenges in patient selection and limit the reproducibility of surgical strategies. Although our study is limited by its retrospective single-center design, the large sample size and a long-term follow-up provide robust evidence supporting the concept that comprehensive treatment in a high-volume center leads to better long-term outcomes. On the other hand, our findings also show that prompt referral to a high-volume center, even after primary tumor resection, does not significantly worsen long-term results. Further prospective studies are warranted to confirm and expand upon these findings.

## Conclusion

The present analysis supports the value of comprehensive, multidisciplinary management in high-volume centers for patients with synchronous CLM. Despite comparable perioperative outcomes, exclusive internal management was associated with a trend toward improved long-term survival after adjustment for key prognostic factors. Centralization of care and early referral to specialized centers remain crucial to ensure optimal patient selection, personalized treatment strategies, and maximization of oncologic outcomes.

## Data Availability

Authors have not planned to make their data available to other researchers.
